# A Novel Additive Manufacturing Method of Cellulose Gel

**DOI:** 10.3390/ma14226988

**Published:** 2021-11-18

**Authors:** Hossein Najaf Zadeh, Daniel Bowles, Tim Huber, Don Clucas

**Affiliations:** 1 College of Engineering, University of Canterbury, Private Bag 4800, Christchurch 8020, New Zealand; djb252@uclive.ac.nz (D.B.); don.clucas@canterbury.ac.nz (D.C.); 2 Biomolecular Interaction Centre, University of Canterbury, Private Bag 4800, Christchurch 8020, New Zealand; Tim.huber@canterbury.ac.nz; 3 School of Product Design, University of Canterbury, Private Bag 4800, Christchurch 8020, New Zealand

**Keywords:** additive manufacturing, cellulose, gel, hydrogel, screen printing, 3D printing, screen additive manufacturing, stencil additive manufacturing

## Abstract

Screen-additive manufacturing (SAM) is a potential method for producing small intricate parts without waste generation, offering minimal production cost. A wide range of materials, including gels, can be shaped using this method. A gel material is composed of a three-dimensional cross-linked polymer or colloidal network immersed in a fluid, known as hydrogel when its main constituent fluid is water. Hydrogels are capable of absorbing and retaining large amounts of water. Cellulose gel is among the materials that can form hydrogels and, as shown in this work, has the required properties to be directly SAM, including shear thinning and formation of post-shearing gel structure. In this study, we present the developed method of SAM for the fabrication of complex-shaped cellulose gel and examine whether successive printing layers can be completed without delamination. In addition, we evaluated cellulose SAM without the need for support material. Design of Experiments (DoE) was applied to optimize the SAM settings for printing the novel cellulose-based gel structure. The optimum print settings were then used to print a periodic structure with micro features and without the need for support material.

## 1. Introduction

In recent years, various approaches based on additive manufacturing (AM), popularly known as 3D printing, of natural materials or biopolymers have been explored to fabricate complex geometries of gels [1,2,3,4]. Although 3D printing technology offers great potential, there exists significant challenges to overcome the limited capabilities of the 3D printing gels, such as part resolution, processing and printing speed, and the lack of diversity in 3D printable biomaterials. One of the classes of materials that has a wide range of applications in bio-engineering is hydrogels [5]. They are highly hydrophilic polymer networks of natural or synthetic origin that are strong enough to absorb and retain a large amount of water.

Polysaccharides are one of the materials that can be turned into hydrogels; the most common being cellulose, chitosan, and agarose. Cellulose, as the most abundant organic compound on Earth, has desirable mechanical and chemical characteristics, including permeability, nontoxicity, biocompatibility, and biodegradability [6]. Moreover, it is an inexpensive and environmentally friendly material. Fabricating hydrogels out of cellulose makes it favorable for many applications, including tissue engineering [7], cell culture [8], drug delivery [9], purification [10], and agrochemicals [11]. Cellulose is neither soluble nor meltable; however, it can be dissolved in a few classes of solvents, such as ionic liquid 1-ethyl-3-methylimidazolium acetate [12], N-methylmorpholine-N-oxide (NMMO) [13], and, the most recently developed, a non-hazardous aqueous solvent of NaOH/urea [14]. Dissolved cellulose in NaOH/urea solvent can form an irreversible gel by the heating or cooling of the solution [15].

The cellulose gel is a potential candidate for shaping using AM [16,17,18,19,20,21]. This behavior was explored previously by locally heating the cellulose solution beyond its gelation point using a focused laser beam. [22]. In our previous work, a cellulose gel formed from dissolving excess cellulose in a solution of urea and sodium hydroxide was 3D printed and the effect of cellulose gel viscosity on 3D printed part resolution was studied [18]. Cellulose gel meets the required printing properties, including shear thinning and post-shear regaining of polymer structural integrity.

Due to the completion of shaping cellulose gel, and when a non-solvent such as water or alcohol is introduced to the gel, the cellulose precipitates, and if water is present, it forms a stable hydrogel [23]. Tunable control over cellulose gelation using physical or chemical crosslinking strategies resulted in a large degree of programmability in behavior [24]. In shaping cellulose/its derivative hydrogel, different potential AM methods have been developed [17,19]. AM methods are broadly classified based on their working principles, such as extrusion, droplet, and laser-based methods. However, limitations to the printability of hydrogels are some of the major constraints that have slowed the application of hydrogel materials. These limitations include part resolution, printing speed, scalability, and creating anatomically correct porous constructs. 

Acknowledging cellulose gel fabrication limitations, we developed a method called screen-additive manufacturing (SAM), or 3D screen-printing, to address the obstacles in shaping hydrogels [6]. In our recent development, we examined how the cellulose gel could be shaped using the developed process. In this method, a computer model of a part was sliced into layers and each layer was used to fabricate a screen. Successive screen-printing of sliced layers resulted in the formation of a three-dimensional object.

Screen-printing of cellulose gel offers a solution for problems associated with other 3D printing methods, including speed of manufacturing, part resolution, and up-scaling. In this study, we present the printability of cellulose gel using the developed SAM process to create three-dimensional objects and determine the print parameters of screen-printing hydrogels using a Design of Experiments (DoE) process. 

In DoE, print setting and process planning can be optimized. In a multi-factor process with different factor levels, DoE can arrange the condition of the experiments. In a full factorial design, all possible combinations of a given set of factors are evaluated simultaneously. Therefore, full factorial designs result in a significant number of experiments. In a 3D printing process that involves three printing factors, such as printing speed, pressure, and temperature, where each factor has five levels, the total number of combinations is 5^3^ = 125. Consequently, due to the high number of trials and the resultant cost, only a few of the possibilities are chosen to keep the number of possible experiments to a minimum [25].

An experimental design that selects only a limited number of data points is called a partial factorial design. Although this shortcut method is well known, there are no general guidelines for its application and no analytical method is available for analyzing the results. Taguchi’s approach complements both of these weaknesses. First, it defines a set of orthogonal arrays (OAs). Second, it offers a standard method for analyzing the results. OA designs are often used in design experiments with multiple-level factors. The symbolic designation for these arrays carries the key information on the size of the experiment. The Taguchi approach measures the quality characteristic differing from the target value by using the signal-to-noise (S/N) ratio. It is also used in the optimum parameter analysis as an alternative to averaging the experimental results [26]. The Taguchi method can play an effective role in identifying the impacts of printing parameters in SAM. A study of the effect of an individual parameter can indicate which parameters have the greatest influence on the performance measure.

## 2. Materials and Methods

### 2.1. Screen-Additive Manufacturing (SAM)

In a typical gel 3D printing process, each structure is fabricated in a layer-by-layer manner and each layer is made of numerous deposited droplets, whereas in the SAM method, each layer is printed in a continuous format, using screens [6]. In this method, a structure is sliced into layers and each layer is used to fabricate a screen. Screens are laser cut out of thin stainless-steel shim and put under tension using a frame. Initially, the cellulose gel needs to be prepared and loaded onto the screens. After the gel is deposited onto a screen, a squeegee is used to spread and push the gel through the voids onto a substrate, known as a print bed. As a result, a layer of cellulose gel is screen-printed onto the print bed. Consequently, the second layer is screen-printed on the previous layer and forms a 3D construct.

**Figure 1:**
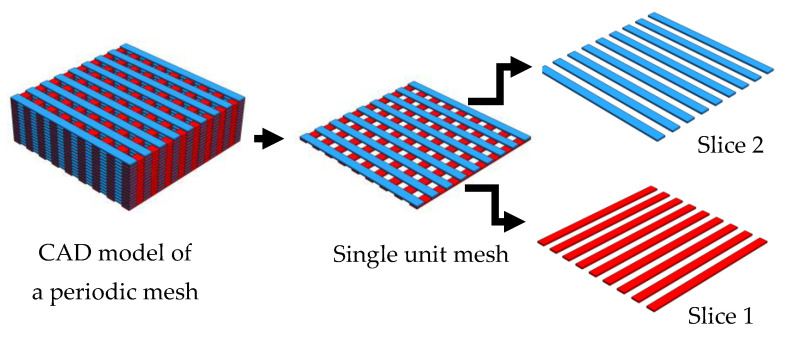
CAD model a periodic structure for SAM.

A screen loading mechanism changes the screen with the next —successive printing layers are made using the sequence of produced screens until a SAM cellulose gel is completed. Screen-printing successive layers of a construct resulted in the fabrication of a 3D structure, as shown in Figure 1.

### 2.2. Screen Features Wall Roughness

One factor that defines SAM part quality is the screen void wall roughness. The wall roughness of the voids fabricated with laser cutting was studied with a DEKTAK 150 surface profilometer (Tucson, AZ, USA). The 3D images of screen voids, walls, and surfaces were taken for further analysis. The surface roughness of cut walls was determined by Ra and Rz, where Ra is the arithmetic average of the absolute values of the roughness profile and Rz is the average maximum height of the profile.

### 2.3. Taguchi L9 Orthogonal Arrays

Maintaining a stable and repeatable part fabrication with the SAM cellulose gel was studied with the use of statistical analysis. In SAM gels, many factors play significant roles, such as material temperature, particle size, hydrophilicity and hydrophobicity, material concentration, and homogeneity; however, the key in this study is not to define and set limitations on all the printing conditions and variables but rather to investigate and demonstrate shaping of the cellulose gel into a periodic micro-feature structure. 

The Taguchi L9 OA was used to determine the optimum and robust SAM setting for the cellulose gel. A Taguchi OA is an experimental design that requires only a fraction of the full-factorial combinations. However, in order to determine factors and factor levels, preliminary trials were conducted. The factors are the variables that can be controlled in the experiment, also known as independent variables. Control factors in SAM are layer thickness, squeegee speed, pressure, printing material formulation, and printing temperature. The printing material formulation was selected based on our recent study of 3D bioprinting of cellulose gel. We found that the optimal cellulose concentration for 3D printing the formulated gel was 20 wt.% cellulose content [18]. The printing process was performed at room temperature (21 ± 2 °C). Therefore, the Taguchi OA was designed with three independent variables. Orthogonal designs allow an estimate of the effect of each factor on the response independently of all other factors. Factors assume only a limited number of possible values, known as factor levels. In the selection of factor levels, over 8000 layers of samples were printed before the Taguchi OA was designed. The factor levels of each independent variable, mentioned above, were determined by measuring the extreme values (maximum and minimum values) in which a sample could be fabricated. The factors and levels, along with the structure of the L9 OA design, are listed in Table 1.

The statistical analysis of the data obtained from lab experiments was performed with analysis of variance (ANOVA) and signal-to-noise (S/N) ratio calculation. ANOVA was used to examine the significance of control factors on SAM cellulose gel. The S/N ratio is a measure of robustness in the Taguchi design that is used to identify control factors that reduce the variability in a process by minimizing the effects of uncontrollable factors (noise factors). Choosing settings with a high S/N ratio minimizes the impact of the noise factors. 

The first variable affecting print quality in SAM gel was considered as the print speed, which is the wiping speed of the gel on a screen. Therefore, three speed levels of 37.5 mm/min, 50 mm/min, and 70 mm/min were selected. The second identified factor impacting the SAM gel process was the applied squeegee force to the screens. The force was transferred to the screens using a pneumatic cylinder with a 10 mm diameter bore. Three levels of 11.8 N (1.5 bar), 15.74 N (2 bar), and 19.67 N (2.5 bar) were chosen for the squeegee force factor. The squeegee blades were made of stainless steel with a dimension of 100 mm width and a thickness of 1 mm. The squeegee angle was set to 60 degrees relative to the screen. The final factor was considered to be printing layer thickness. The screen thickness had a significant effect on the print layer thickness. The layer thickness was determined by the vertical increment of the stage. The screens were made out of stainless-steel grade 304 with 0.08 mm thickness. The screens were tensioned using a custom-designed frame with threaded shafts. 

The optimum levels of each factor can be determined by applying a standard analysis. The Taguchi L9 OA trials were completed by conducting multi-response experiments on screen-printing a cube. Based on the experimental design with the L9 OA, nine combinations of experiments were performed twice. Replication was done to avoid probable errors, as well as to consider the influence of uncontrollable factors in the laboratory (noise).

**Figure 2:**
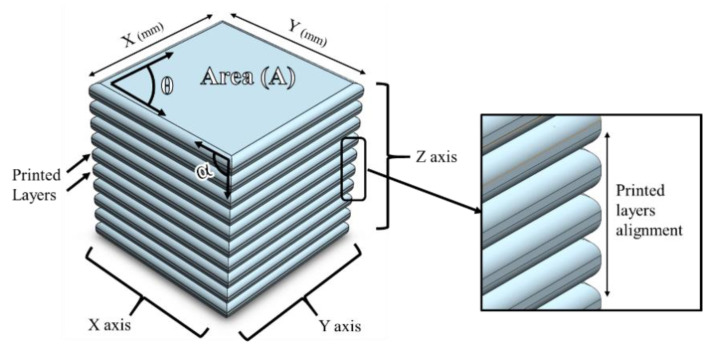
CAD model of printing cube and the measuring parameters.

**Figure 3:**
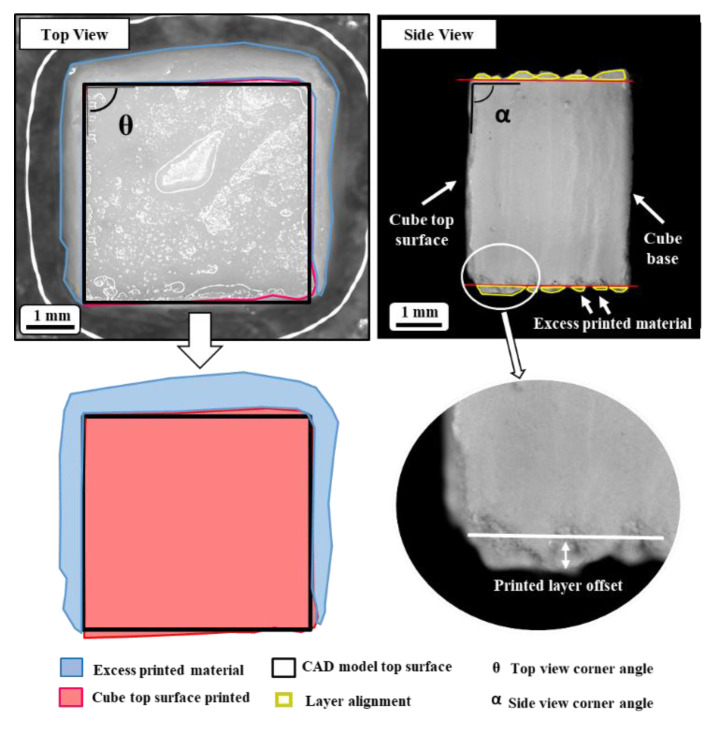
Printed cube top and side view, 5 mm × 5 mm cube, 40 layers.

The CAD model of the screen-printed cube and the measuring parameters are displayed in Figure 2. The selected inputs for the statistical analysis were the deviation of the screen-printed cube top surface (A) from the CAD model and the precision of successive layers aligning on top of each other. Figure 3 shows a printed sample, in which the highlighted area corresponds to the deviation from the CAD model. It should be noted that in an ideal SAM gel part, there should not be any excess printed material. A microscope equipped with a camera (Canon, Tokyo, Japan) was used to capture the images of the top and side views of the samples, and ImageJ Fiji software (National Institutes of Health, Bethesda, US) was used to analyze the results.

In the analysis of the results, the target value of “smaller is best” was selected to determine the S/N ratio. The experiments aimed to fabricate the nearly cubic shape samples. The S/N ratio for a target value of “the smaller the better” could be calculated using Equation (1):

S/N = −10 × log (Σ (Y2)/n)(1)

where Y = responses for the given factor level combination and n = number of responses in the factor level combination. 

The L9 OA experiments were performed on a cube with a dimension of 5 mm by 5 mm cross-section. The cube height varied based on the selection of layer thickness factor. For each trial of L9 OA, 40 successive layers were printed. The measured data were analyzed using Minitab software (Minitab Inc., State College, PA, USA). 

The measured data were subjected to the Minitab statistical software package to predict the nearly cubic shape cellulose gel part at proposed optimum SAM settings. A confirmation experiment was also performed to check the accuracy of proposed optimum SAM settings. In the confirmation test, a cube with 5 mm × 5 mm cross-section and 100 layers height was screen-printed.

**Figure 4:**
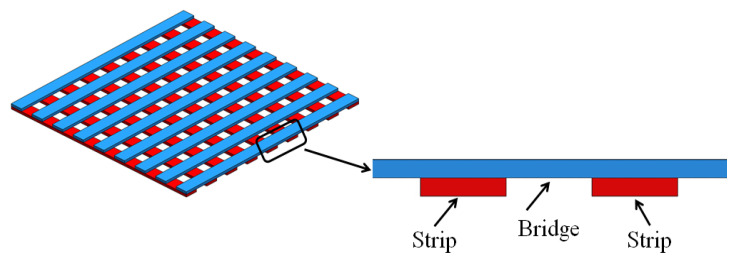
CAD model of a mesh showing strips and the bridges.

The final part design to screen-print was a more complex geometry. In the fabrication of a mesh structure, as displayed in Figure 4, two screens were made out of two slices of the CAD model. Printing sequential screens alternately led to the fabrication of periodic mesh structures.

### 2.4. Screen-Additive Manufacturing Scaffold-Free

For complex geometries, such as the microchannel periodic structure illustrated in Figure 1 and Figure 4, removing support materials would be a challenging task. Therefore, the capability of SAM scaffold-free cellulose gel was investigated. A periodic structure with 500 μm channels was printed to measure the overhangs and bridges. A bridge is a 90° link that is supported from at least two points, as shown in Figure 4.

### 2.5. Preparation of Materials

A solvent consisting of 81 wt.% DI water, 12 wt.% urea (Sigma-Aldrich, BioUltra, purity of 99.5%), and 7 wt.% sodium hydroxide NaOH (The Sourcery, Christchurch, NZ, purity of 99%) was mixed using a Velp Scientifica DLS overhead stirrer (Usmate Velate, Italy) at 100 rpm. After mixing was completed, the mixture was heated to approximately 50 °C using a Philips (Amsterdam, Netherlands) 2000 W microwave for 30 s. Cellulose powder (Sigmacell, type 20, 20 μm, Sigma-Aldrich, St. Louis, MI, USA) 20 wt.% was added to the solvent mixture and stirred at 100–120 rpm for 2 min. The formulation was then put in a freezer set to −18 °C for 45 min to dissolve the cellulose. Subsequently, the solution was stored in a refrigerator at 5 °C until used by the screen AM machine. The L9 OA tests were completed by SAM, with 40 successive layers. Results were analyzed before proceeding to SAM with a more complex geometry. 

## 3. Results and Discussion

One of the main parameters in SAM gel is viscoelasticity. The formulated cellulose solution/gel behaves as a shear-thinning liquid [27], which makes it ideal for the SAM process. In the SAM process, the gel underwent shear stress from the force applied by a squeegee and dispersed from the screen voids. Shear force breaks down the gel into fine fragments and, upon removal of the force, the gel retains its shape [18]. This phenomenon is due to the presence of hydrogen groups in the dissolved and suspended portions of cellulose and also the interparticle forces between the dispersed cellulose particles and potentially gel fragments [28]. The dispersed cellulose particles result from added cellulose powder to the solution. The extra cellulose powder in the solution acts as a cross-linking agent and strengthens gels [29].

After completion of L9 OA, results from the measured tests showed that, by changing the print settings, the deviated inward/outward top surface area from the CAD model cube (excess top surface area) is varied between 6.48 mm^2^ to 22.11 mm^2^, and layer alignment offset varied from 0.11 mm to 0.35 mm. The L9 OA trials and the measured results are presented in Table 2.

In the Taguchi optimization procedure, the analysis of the experimental data using the ANOVA gave an output that was statistically significant in the outcome of the optimum SAM conditions. The analysis of variance for the S/N ratio showed that layer thickness had significant effects on the SAM process with a contribution percentage of more than 92.6%. The R-squared (coefficient of determination) was calculated to 81%. which indicated that the model explains all the variability of the response data around its mean. The control factors “layer thickness”, “squeegee speed”, and “squeegee pressure” with F-ratio values of 12.43, 1.75, and 5.97 had the least experimental errors, respectively. 

**Figure 5:**
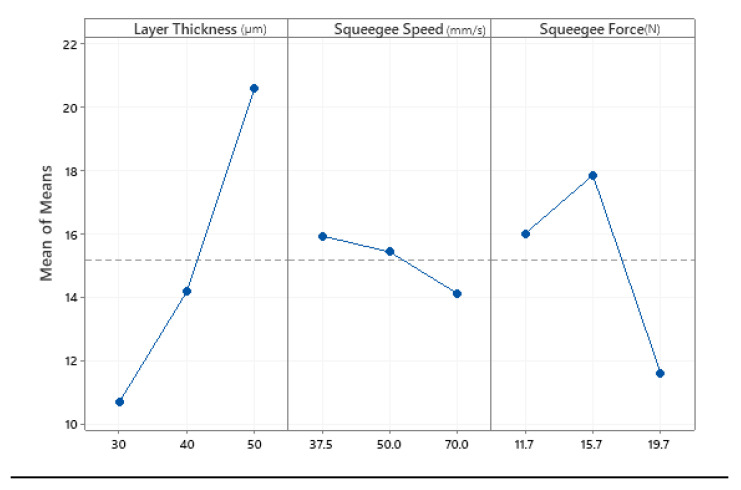
Main effect of the plot for mean values.

**Figure 6:**
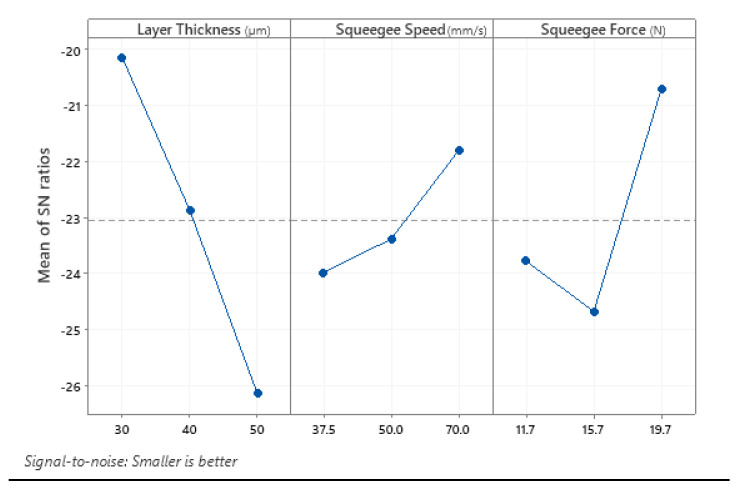
Main effects plot for S/N ratios.

The main effect plot for the mean value and S/N ratio of the samples were plotted, as shown in Figure 5 and Figure 6. Since the target value was chosen to be “smaller is better”, the optimum condition for each factor was the level that gives the smallest point of means of each plot for measured parameters of excess top surface area and layer alignment offset. Results from the main effects plot of means indicated that the optimum SAM settings were achieved with the combination of 30 μm printing layer thickness, with a printing speed (squeegee speed) of 70 mm/min and a squeegee force of 19.74 N (gauge pressure of 2.5 bar).

Using the L9 OA, we conducted a confirmation test in which similar results were obtained. Table 3 shows the confirmation experiment result. 

Table 4 shows the response table for the S/N ratio of the deviated top surface area. The greater difference (Delta Δ) value for the average S/N ratio indicates that the control factors have a greater substantial effect on the S/N ratio. Therefore, layer thickness, squeegee speed, and squeegee force have the most effects on SAM cellulose gel parts, respectively. 

For the SAM gel process, an important parameter that affects the part quality is layer alignment. During the layer on the layer printing process, layers could be misaligned, resulting in uneven wall surfaces and poor print quality. For applications such as 3D printing vessel constructs for ultrasound phantoms and biomimetic vascularization, the smoothness of the gel constructs is a critical parameter [30,31]. Several factors affect the alignment of printed layers, such as screen positioning on a print bed, printing material volume, and complete gel release from the screen voids.

The positioning of screens on the print bed needs to be controlled in successive layer prints. Misalignment can be caused by a variation in the exact positioning of screens relative to the print bed. As the screens were positioned against a hard surface, misalignments were minimized. The tolerance for screen positioning in SAM is considered to be less than 0.01 mm. Another parameter contributing to printing layer misalignment is the volume of released material from the screens; excessive or insufficient printing material would cause uneven printed wall surfaces. The squeegee force, squeegee speed, and material of the squeegee blade determine the volume of the material to be screen-printed. Lastly, the release of gel from the screen features/voids needs to be monitored. Surface roughness of cut features on the screens and cut profiles, such as parallel/tapered walls, can influence the complete release of material from the screens [6].

**Figure 7:**
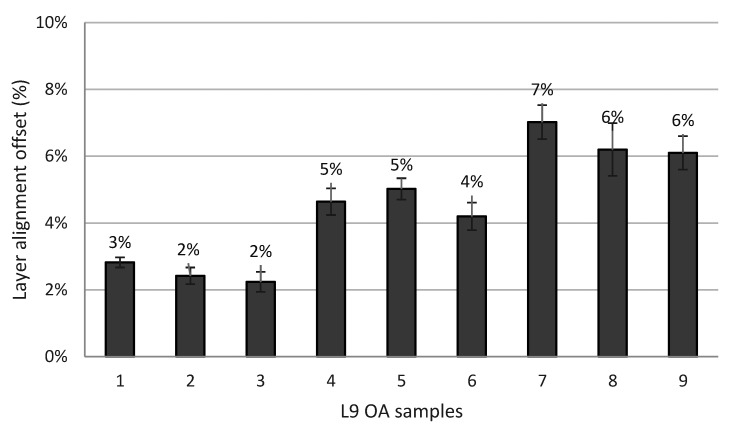
Layer alignment offset percentage for L9 OA.

The precision of screen-printed layers is measured and presented in Figure 7. Results indicated that the minimum layer offset belongs to test number three, with an offset layer of 110 μm, which accounts for 2% misalignment, where the worst layers alignment belongs to test number seven with 351 μm layers offset, equivalent to 7% offset. Considering the statistical analysis of the DoE process, the predicted SAM settings resulted in high shape fidelity and, ultimately, fabrication of features with a smooth wall surface. Analysis of the results indicated that, by increasing the layer thickness, the layer misalignment increased. 

**Figure 8:**
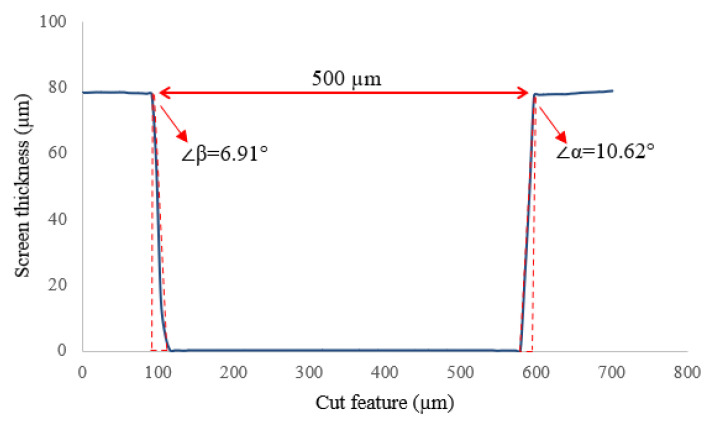
Aperture profile of a laser-cut screen with wall angles of β = 6.91° and α = 10.62°, the aperture width 500μm and stencil thickness of 80 μm.

In the SAM process, the screens were located in a precise position to avoid layer misalignment. In addition, the surface roughness and cut profile of screens were determined and presented in Figure 8. Ra, for the laser cut edge without any cut surface treatment, was found to be 6 μm, and Rz was measured at 12 μm. Surface treatment can be used to achieve a smoother cut wall surface. For example, the inside aperture walls of electro-polished laser-cut screens are smoother than those on laser-cut screens that have not been electro-polished [32]. Thus, the former releases a higher volume of printing material than the latter with a similar area ratio.

The observed layer misalignment is the result of print material bleeding from the edge of the pattern on the screen. The bleeding is referred to as the excess material pushed through the screen openings. It was found that the increase in the squeegee speed reduces the effect of bleeding material and the misalignments. 

The aperture cut angles were measured β = 6.91° and α = 10.62° for each sidewall. This tapered shape cut could prevent the complete release of the printing gel from the screen aperture. Therefore, it is necessary to clean the screens after printing each layer to avoid damaging the printed layers. 

### 3.1. 3D Printing Overhangs 

Complex geometry part fabrication in AM relies heavily on support material to ensure that the printed part is stable and has sufficient support [33]. Generally, features exceeding 45° require support structures. Printing structures with support material encounter the issue of support material removal, which is a very time-consuming process that generates waste material and consequently costs. The problem becomes worse when dealing with microfeatures and microchannels, such as those in complex geometries. 

**Figure 9:**
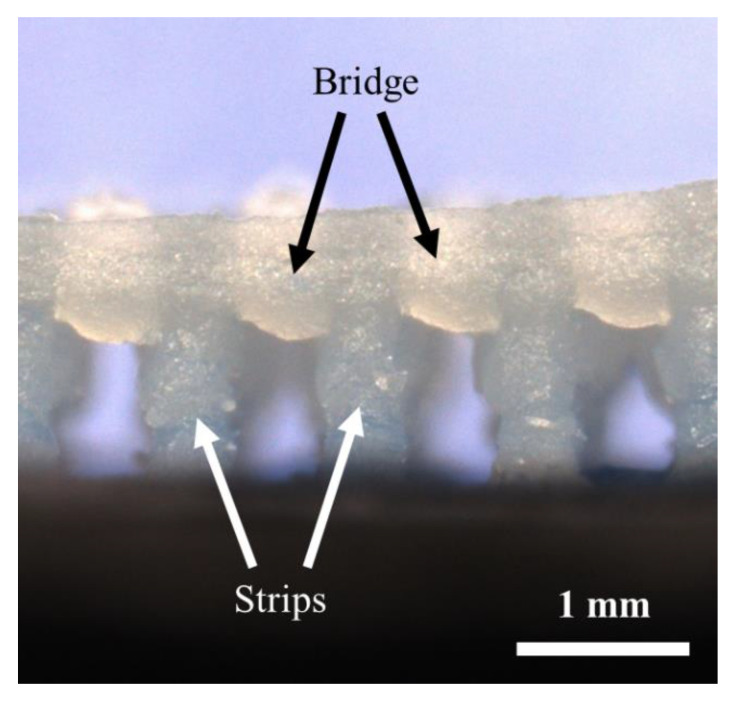
Side view of a SAM cellulose gel, showing strips and bridges.

Bridges and overhangs that lack enough support are prone to curling, delamination, sagging, and collapsing during screen-printing a periodic mesh construct. Therefore, the ability of screen-print bridges and overhangs without the need for supporting structure were measured. Figure 9 displays the side view of mesh construction that contains bridges and overhangs. The strips were screen-printed by repeating the print 20 times of the first screen, followed by printing the second screen, which resulted in printing bridges without any support structure. The shear force applied from the squeegee reduced the viscosity of the printing gel and caused the flow of the gel through the screen features. Upon removal of the squeegee force, the gel regained its strength and formed a viscose gel. The cellulose gel physical crosslinking and the inter-particle forces were sufficient to hold the bridging structures.

Since more than one screen was used in printing the mesh structure, the tension of the screens was considered an important factor in achieving a consistent layer thickness. It was found that an approximate same screen tension was needed when printing a part that required more than two screens. The SAM parts could fail due to variations in screen tension. A low screen tension could cause the screen to bend excessively, while a high tension could cause partial material release during the printing process. The effect of screen tension on part quality should be investigated in other research. 

### 3.2. Confirmation Experiments

**Figure 10:**
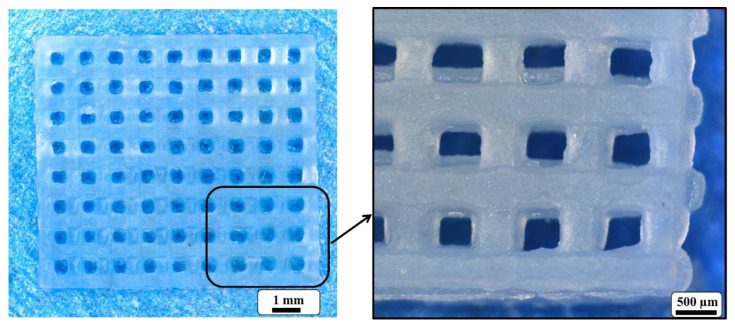
Test prints of a mesh using optimal settings for SAM from the Taguchi analysis of the print parameters.

Validation of the SAM optimization was produced by carrying out a confirmation experiment, implementing the assigned input parameters achieved from L9 OA. A mesh geometry cellulose gel was constructed and presented in Figure 10.

**Figure 11:**
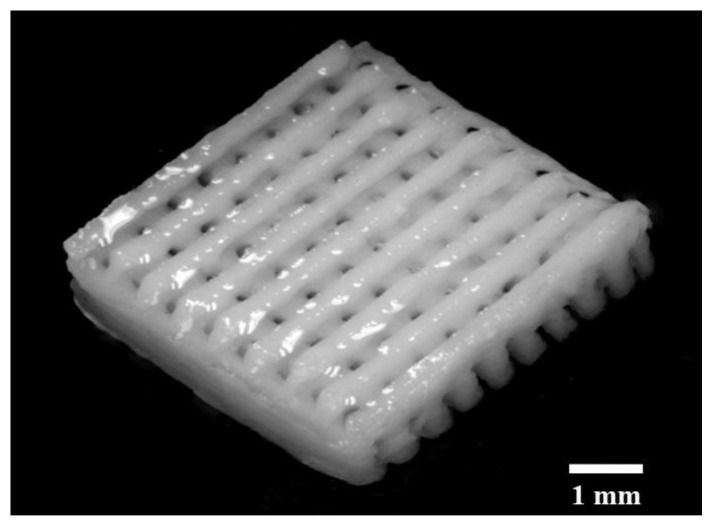
SAM a mesh structure using cellulose gel.

In order to regenerate the SAM cellulose gel, the printed part was placed in a DI water bath to replace the solvent in the gel with water and form a regenerated cellulose hydrogel [34]. The image of water regenerated cellulose is shown in Figure 11. The regeneration of cellulose gel, by washing it, lead to pore contraction. Regeneration time is dependent on the kinetics of cellulose solvent release into the bath [35], which is dependent on regenerating conditions and screen-AM part geometry. 

The regenerated cellulose was swollen in water, and once the regeneration was completed, the printed layers adhered and no delamination occurred.

## 4. Conclusions

The Taguchi method for SAM cellulose gel was applied in order to understand the process and achieve the best shape fidelity gel fabrication. We have determined the effect of three efficient independent parameters in the process of SAM. The optimum print settings for the printing of 20 wt.% cellulose gel was considered to be 19.7 N of squeegee force, 70 mm/min squeegee speed, and 30 μm layer thickness for a screen with an 80 μm thickness. It was found that layer thickness, squeegee speed, and squeegee force have the greatest to the least effects on SAM cellulose gel parts, respectively. 

According to predicted and calculated results, cellulose gel can be direct SAM without the need of support material, which makes it a suitable method for efficiently manufacturing micro size periodic structures. The optimized SAM settings were examined in confirmation part production. The confirmation test indicated that the predicted SAM factor levels could result in the fabrication of intricate cellulose gel parts.

## Figures and Tables

**Table 1:** A list of print trials at varying settings for layer thickness, squeegee speed, and squeegee pressure.

Trial	Layer Thickness (µm)	Squeegee Speed (mm/s)	Squeegee Force (N)
1	30	37.5	11.8
2	30	50	15.74
3	30	70	19.67
4	40	37.5	15.74
5	40	50	19.67
6	40	70	11.8
7	50	37.5	19.67
8	50	50	11.8
9	50	70	15.74

**Table 2:** Results of the L9 OA tests and measurements for the 5 mm by 5 mm cube.

Test No.	Layer Thickness (µm)	Squeegee Speed (mm/s)	Squeegee Force (N)	Excess Top Surface Area (mm^2^)	Layer Alignment Offset (mm)
1	30	37.5	11.7	13.74	0.14
2	30	50	15.7	11.81	0.12
3	30	70	19.7	6.48	0.11
4	40	37.5	15.7	18.03	0.23
5	40	50	19.7	12.33	0.25
6	40	70	11.7	12.15	0.21
7	50	37.5	19.7	15.99	0.35
8	50	50	11.7	22.11	0.31
9	50	70	15.7	23.68	0.30

**Table 3:** Confirmation of predicted SAM settings.

Layer Thickness (μm)	Speed (mm/s)	Force (N)	Excess Top Surface Area (mm^2^)	Layer Alignment Offset (%)
30	70	19.7	5.21	2

**Table 4:** Response table for S/N ratio.

Level	Layer Thickness	Squeegee Speed	Squeegee Force
1	−20.15	−23.99	−23.78
2	−22.88	−23.39	−24.69
3	−26.15	−21.80	−20.71
Delta	6.00	2.18	3.98
Rank	1	3	2

## Data Availability

The data presented in this study are available upon request from the corresponding author.

## References

[1] Puertas-Bartolomé M., Mora-Boza A., García-Fernández L. (2021). Emerging Biofabrication Techniques: A Review on Natural Polymers for Biomedical Applications. Polymers.

[2] Chimene D., Lennox K.K., Kaunas R.R., Gaharwar A.K. (2016). Advanced Bioinks for 3D Printing: A Materials Science Perspective. Ann. Biomed. Eng..

[3] Gopinathan J., Noh I. (2018). Recent trends in bioinks for 3D printing. Biomater. Res..

[4] Malda J., Visser J., Melchels F.P., Jüngst T., Hennink W.E., Dhert W., Groll J., Hutmacher D.W. (2013). 25th Anniversary Article: Engineering Hydrogels for Biofabrication. Adv. Mater..

[5] Park H., Park K. (1996). Hydrogels in Bioapplications. ACS Symp. Ser..

[6] Najaf Zadeh H. (2019). Mass Fabrication of High-Resolution Hydrogels by a High-Speed Process Using a Thermal 3D Screen Printing Method.

[7] Mohanty S., Larsen L.B., Trifol J., Szabo P., Burri H.V.R., Canali C., Dufva M., Emnéus J., Wolff A. (2015). Fabrication of scalable and structured tissue engineering scaffolds using water dissolvable sacrificial 3D printed moulds. Mater. Sci. Eng. C.

[8] Bhattacharya M., Malinen M.M., Laurén P., Lou Y.-R., Kuisma S.W., Kanninen L., Lille M., Corlu A., GuGuen-Guillouzo C., Ikkala O. (2012). Nanofibrillar cellulose hydrogel promotes three-dimensional liver cell culture. J. Control. Release.

[9] Ranjha N.M., Qureshi U.F. (2014). Preparation and characterization of crosslinked acrylic acid/hydroxypropyl methyl cellulose hydrogels for drug delivery. Int. J. Pharm. Pharm. Sci..

[10] Liu J., Chu H., Wei H., Zhu H., Wang G., Zhu J., He J. (2016). Facile fabrication of carboxymethyl cellulose sodium/graphene oxide hydrogel microparticles for water purification. RSC Adv..

[11] Pang L., Gao Z., Feng H., Wang S., Wang Q. (2019). Cellulose based materials for controlled release formulations of agrochemicals: A review of modifications and applications. J. Control. Release.

[12] Tan X., Chen L., Li X., Xie F. (2019). Effect of anti-solvents on the characteristics of regenerated cellulose from 1-ethyl-3-methylimidazolium acetate ionic liquid. Int. J. Biol. Macromol..

[13] Zhao H., Kwak J., Wang Y., Franz J., White J., Holladay J. (2007). Interactions between cellulose and N-methylmorpholine-N-oxide. Carbohydr. Polym..

[14] Cai J., Zhang L. (2005). Rapid Dissolution of Cellulose in LiOH/Urea and NaOH/Urea Aqueous Solutions. Macromol. Biosci..

[15] Cai J., Zhang L. (2006). Unique Gelation Behavior of Cellulose in NaOH/Urea Aqueous Solution. Biomacromolecules.

[16] Zadeh H.N., Huber T., Nock V., Fee C., Clucas D. (2020). Complex Geometry Cellulose Hydrogels Using a Direct Casting Method. Bioengineering.

[17] Wang Q., Sun J., Yao Q., Ji C., Liu J., Zhu Q. (2018). 3D printing with cellulose materials. Cellulose.

[18] Huber T., Zadeh H.N., Feast S., Roughan T., Fee C. (2020). 3D Printing of Gelled and Cross-Linked Cellulose Solutions; an Exploration of Printing Parameters and Gel Behaviour. Bioengineering.

[19] Dai L., Cheng T., Duan C., Zhao W., Zhang W., Zou X., Aspler J., Ni Y. (2019). 3D printing using plant-derived cellulose and its derivatives: A review. Carbohydr. Polym..

[20] Wang J., Chiappone A., Roppolo I., Shao F., Fantino E., Lorusso M., Rentsch D., Dietliker K., Pirri C.F., Grützmacher H. (2018). All-in-One Cellulose Nanocrystals for 3D Printing of Nanocomposite Hydrogels. Angew. Chem. Int. Ed..

[21] Cheng Y., Shi X., Jiang X., Wang X., Qin H. (2020). Printability of a Cellulose Derivative for Extrusion-Based 3D Printing: The Application on a Biodegradable Support Material. Front. Mater..

[22] Huber T., Clucas D., Vilmay M., Pupkes B., Stuart J., DiMartino S., Fee C. (2018). 3D Printing Cellulose Hydrogels Using LASER Induced Thermal Gelation. J. Manuf. Mater. Process..

[23] Wang S., Lu A., Zhang L. (2016). Recent advances in regenerated cellulose materials. Prog. Polym. Sci..

[24] Zhao D., Huang J., Zhong Y., Li K., Zhang L., Cai J. (2016). High-Strength and High-Toughness Double-Cross-Linked Cellulose Hydrogels: A New Strategy Using Sequential Chemical and Physical Cross-Linking. Adv. Funct. Mater..

[25] Roy R.K. (2010). A Primer on the Taguchi Method.

[26] Montgomery D.C. (2017). Design and Analysis of Experiments.

[27] Huber T., Starling K., Cen W. (2016). (Samantha); Fee, C.; Dimartino, S. Effect of Urea Concentration on the Viscosity and Thermal Stability of Aqueous NaOH/Urea Cellulose Solutions. J. Polym..

[28] Wilson S.A., Cross L.M., Peak C.W., Gaharwar A.K. (2017). Shear-Thinning and Thermo-Reversible Nanoengineered Inks for 3D Bioprinting. ACS Appl. Mater. Interfaces.

[29] Huber T., Feast S., DiMartino S., Cen W., Fee C. (2019). Analysis of the Effect of Processing Conditions on Physical Properties of Thermally Set Cellulose Hydrogels. Materials.

[30] Nikitichev D.I., Barburas A., McPherson K., Mari J.M., West S.J., Desjardins A.E. (2016). Construction of 3-Dimensional Printed Ultrasound Phantoms With Wall-less Vessels. J. Ultrasound Med..

[31] Li L., Qin S., Peng J., Chen A., Nie Y., Liu T., Song K. (2020). Engineering gelatin-based alginate/carbon nanotubes blend bioink for direct 3D printing of vessel constructs. Int. J. Biol. Macromol..

[32] Burgess M.R., Coleman W.E. (2007). Electroformed vs. Laser-cut: A Stencil Performance Study. SMT-TULSA-.

[33] Ngo T.D., Kashani A., Imbalzano G., Nguyen K.T.Q., Hui D. (2018). Additive manufacturing (3D printing): A review of materials, methods, applications and challenges. Compos. Part B Eng..

[34] Zhang L., Ruan D., Gao S. (2002). Dissolution and regeneration of cellulose in NaOH/thiourea aqueous solution. J. Polym. Sci. Part B Polym. Phys..

[35] Sescousse R., Budtova T. (2009). Influence of processing parameters on regeneration kinetics and morphology of porous cellulose from cellulose–NaOH–water solutions. Cellulose.

